# Association of quantitative analysis of intratumoral reduced E-cadherin expression with lymph node metastasis and prognosis in patients with breast cancer

**DOI:** 10.1038/s41598-023-37012-4

**Published:** 2023-06-27

**Authors:** Xiangyue Meng, Michi Morita, Sayaka Kuba, Hiroko Hayashi, Ryota Otsubo, Megumi Matsumoto, Kosho Yamanouchi, Kazuma Kobayashi, Akihiko Soyama, Masaaki Hidaka, Kengo Kanetaka, Takeshi Nagayasu, Susumu Eguchi

**Affiliations:** 1 Department of Surgery, Nagasaki University Graduate School of Biomedical Sciences, 1-7-1 Sakamoto-Machi, Nagasaki, 852-8501 Japan; 2 Department of Pathology, Nagasaki University Graduate School of Biomedical Sciences, Nagasaki, Japan; 3 Department of Surgical Oncology, Nagasaki University Graduate School of Biomedical Sciences, Nagasaki, Japan

**Keywords:** Breast cancer, Tumour biomarkers

## Abstract

Loss of E-cadherin expression is a poor prognostic factor in patients with breast cancer. Breast cancer cells co-cultured with adipocytes reportedly promote E-cadherin attenuation and tumor progression. The current study aimed to investigate the association of reduced E-cadherin expression with adipose tissue invasion (ATI) and prognosis in breast cancer. Surgical specimens were collected from 188 women with invasive ductal carcinoma of the breast who had undergone surgery without neoadjuvant treatment. We compared E-cadherin expression in ATI and invasive front (IF) using immunohistochemistry with ImageJ. Reduced E-cadherin expression was detected not only in the ATI area but also in the IF, and the degree of reduced E-cadherin expression was positively correlated with both areas. In patients with lymph node metastasis compared to those without, E-cadherin expression was reduced and this reduction was associated with poor recurrence-free survival. We concluded that E-cadherin expression is reduced not only at the ATI area but also at the IF of the tumor. Reduced E-cadherin expression is a clear prognostic factor for breast cancer. Hence, future research is warranted for establishing an objective and quantitative E-cadherin staining assay that will allow clinical use of E-cadherin as a prognostic factor.

## Introduction

Breast cancer affects one in eight women during their lifetime and leads to more than 40,000 deaths in the United States annually^1, 2^. E-cadherin is widely present in epithelial cells, including normal mammary glands. Along with actin, E-cadherin functions to maintain the integrity of epithelial tissues. When E-cadherin expression is disrupted by tumors, intercellular junctions are altered, and subsequently cell migration ability, tumor invasion, and metastasis are increased^3^. In invasive ductal carcinoma of the breast, E-cadherin strongly stained the extracellular membrane, but downregulation of E-cadherin worsened overall survival (OS) as well as disease-free survival (DFS)^4^. In the above-mentioned studies, reduced E-cadherin expression was determined only subjectively; E-cadherin expression was not quantified and no comparison was conducted between the reduced expression and prognosis.

Most of the breast tissue is adipose tissue, and adipose surrounding a tumor is the key component of breast cancer progression^5, 6^. In general, obesity is considered to be one of the risk factors for breast cancer. Pathologic evidence of fat invasion, such as scattered invasion into fat tissues and the invasive length of fat invasion, has been reported to be related to poor prognosis^7^. Yamaguchi et al. reported adipose tissue invasion (ATI) to be an independent factor influencing lymph node (LN) metastasis, and patients with ATI had poorer DFS than those without ATI^8^. Moreover, they had reported ATI-positive patients with triple-negative breast cancer (TNBC) to have significantly worse DFS than luminal patients, although not so for ATI-negative patients^9^. Adipocytes are the primary cellular components comprising the breast cancer microenvironment, may cause the progression of epithelial–mesenchymal transition, and can become more aggressive in an animal models ^10^. The mechanism by which E-cadherin expression is attenuated in breast cancer is not clear; however, tumor-fat interactions may be a contributing factor.

We hypothesized that E-cadherin expression is reduced by ATI of breast cancer cells, leading to LN metastasis and the development of metastasis. Therefore, the two objectives of this study were to clarify whether reduced E-cadherin expression is specific to the ATI area and to elucidate the possible relationship between reduced E-cadherin expression and prognosis.

## Material and methods

### Patients

This retrospective study was approved by the Nagasaki University Hospital Clinical Research Ethics Committee (Approval Number, 20,021,001), and the requirement for informed consent was waived. The study was performed in accordance with ethical standards, as described in the 1964 Declaration of Helsinki and its later amendments. We reviewed 188 patients with invasive ductal breast cancer who had undergone surgery from January 2012 to December 2015 at Nagasaki University. The differentiation between lobular carcinoma and ductal carcinoma was performed morphologically, as described in the WHO guidelines^11^. The presence of cellular cohesion, such as cord-like or nest-like patterns, was diagnosed as ductal carcinoma, whereas a lack of typical cellular cohesion, such as linear arrangement or target-shaped pattern, was diagnosed as lobular carcinoma. Patients with bilateral breast cancer or those who underwent neoadjuvant chemotherapy were excluded. All surgical specimens were formalin-fixed and paraffin-embedded; 4 µm-thick-sections were stained with hematoxylin–eosin, and slides were re-reviewed retrospectively by two independent investigators to distinguish between the presence and absence of ATI. We defined breast cancer subtypes as follows: luminal (hormone receptor positive/human epidermal growth factor receptor HER2 negative), HER2 (any hormone receptor/HER2 positive), and TNBC (hormone receptor negative/HER2 negative).

### Immunohistochemistry

For immunohistochemistry, 4 µm-thick-paraffin sections of the surgically-obtained specimen were heated in ethylenediaminetetraacetic acid (EDTA) buffer using an autoclave for antigen retrieval, incubated in peroxidase-blocking solution (Dako REAL S2023, Dako, Japan) for 10 min at room temperature (RT; 20–25 °C [68–77 °F]) to quench endogenous peroxidase activity, and then blocked for 15 min at RT for non-specific binding (Dako Protein Block Serum-Free X0909). Blocked sections were incubated with E-cadherin antibody (monoclonal mouse Anti-Human NCH-38 ready-to-use, Dako, Japan) for 2 h at RT. Sections were then incubated in the secondary antibody horseradish peroxidase (HRP)-conjugated rabbit anti-mouse (Dako REAL EnVision) for 15 min at RT. HRP-conjugated secondary antibody binding was visualized using the Dako Liquid DAB + Substrate Chromogen System (Dako), and the nuclei were stained with hematoxylin. Bright-field images were captured using an optical microscope (Olympus BX53, Shinjuku, Tokyo, Japan).

### Definitions of ATI and IF

**Figure 1:**
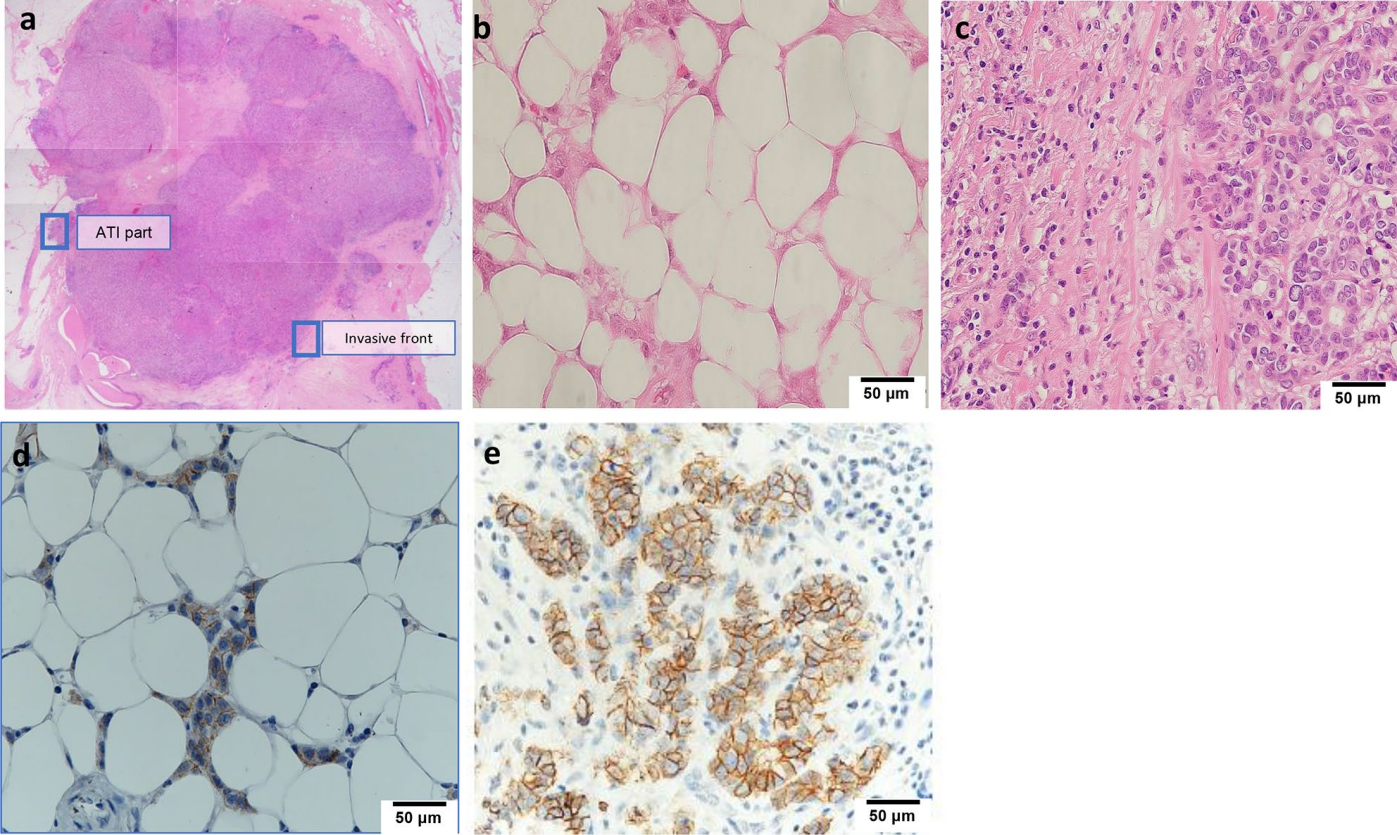
The location of each area in hematoxylin–eosin (HE) staining (**a**) Representative microscope image showing HE staining of intratumor E-cadherin in adipose tissue invasion (ATI) and (**b**) invasive front (IF) (**c**) immunohistochemical staining of intratumor E-cadherin in ATI and (**d**) IF (**e**) Magnified view (40x) of HE staining and immunohistochemical staining of intratumor E-cadherin in ATI and IF shown in (**b**) (**c**) and (**d**) (**e**).

The location of ATI and the invasive front (IF) are shown in Fig. 1a. ATI was defined as the direct presence of more than 20 cancer cells in adipose tissue without intervening fibrous tissues, in accordance with a previous report (Fig. 1b)^8^. The IF in the mammary gland without contacting the adipose tissue was defined as the IF area (Fig. 1c). The control area was defined as the area with the highest intensity in each case. To distinguish between the presence and absence of ATI, all surgical specimens were reviewed by two independent investigators. ATI and IF were evaluated in cases with ATI, and IF was only evaluated in cases without ATI.

### Evaluation of reduced E-cadherin expression

Three independent investigators reviewed the slides and evaluated the two above-mentioned sites. The weakest-stained areas were captured in the high power field for each site. Expression of E-cadherin was quantified using ImageJ software (National Institutes of Health, USA and Fiji)^12^ as follows: First, all tumor clusters were traced manually in the captured areas (Fig. S1). Second, each cluster was numbered, and the E-cadherin intensity and area of each chosen cluster were measured using ImageJ. Third, attenuation of E-cadherin was calculated using the following formula: [1−{Sum (area × intensity of each cluster)/whole area}/control intensity] × 100 (Fig. S1). The highest intensity area in each case was used as the control. The maximum value of E-cadherin attenuation in each area was used as a representative value to examine the relationship between E-cadherin levels and ATI and LN metastasis.

### Statistical analysis

Baseline characteristics and clinicopathological features are described as frequencies for categorical variables and median, and interquartile ranges for quantitative variables. Associations between variables were assessed using the chi-square test for categorical variables and the Mann–Whitney U test for quantitative variables. Correlation for E-cadherin attenuation at ATI and IF was analyzed using the Pearson correlation coefficient. Univariate analysis was performed to identify factors associated with LN metastasis, and factors showing significance were included in the multivariate analysis. Logistic regression analysis was used in the multivariate analysis of LN metastasis and the reduced E-cadherin expression. Recurrence-free survival (RFS) was defined as the absence of any breast cancer recurrence. The events were defined as follows: RFS, all breast cancer recurrences and all-cause mortality; DFS, RFS plus contralateral breast cancer or second cancer; and OS, all-cause mortality^13^. An optimal cut-off score of E-cadherin was calculated to predict RFS by receiver operating characteristic analysis using the presence or absence of overall recurrence at 5 years as outcomes. Survival curves of RFS were constructed using the Kaplan–Meier analysis and log-rank test. All statistical analyses were performed using IBM SPSS software version 26 (Armonk, NY, USA: IBM Corp) and Prism version 8 (GraphPad, San Diego, CA, USA). *P*-values < 0.05 were considered statistically significant.

### Ethical approval

*Approval of the research protocol by an Institutional Reviewer Board*: The study was approved by the Nagasaki University Hospital Clinical Research Ethics Committee (approval number, 20,021,001) and was performed in accordance with the ethical standards described in the 1964 Declaration of Helsinki and its later amendments.

### Informed consent

The need for informed consent was waived owing to the retrospective nature of the study.

## Results

### Clinicopathological characteristics of patients with invasive ductal breast cancer

The study included 188 participants aged 31–92 years (median, 59 years). The median tumor size was 1.6 cm, 69.7% of patients were ATI positive, and 33.5% of patients had LN metastasis (Table 1). The number of patients in each breast cancer subtype was as follows: luminal, 138; HER2, 37; and TNBC, 12.

**Table 1:** Clinicopathological characteristics of patients with invasive breast cancer.

Characteristics	Cases (n = 188)
Median age (range)	59 (31–92)
Tumor size (cm)	1.6 (0.3–6.5)
ATI positive/negative	131 (69.7%)/57 (30.3%)
Nuclear grade 1/2/3	81 (43.1%)/37 (19.7%)/70 (37.2%)
Lymphovasucuar invasion positive/negative	94 (50.0%)/94 (50.0%)
Vessel invasion positive/negative	115 (61.2%)/73 (38.8%)
ER positive/negative	170 (90.4%)/18 (9.6%)
PgR positive/negative	152 (80.8%)/35 (18.6%)^a^
HER2 positive/negative	37 (19.6%)/150 (79.8%)^a^
Subtype Luminal^1^/HER2^2^/TN^3^	138 (73.4%)/37 (19.7%)/12 (6.4%)
Lymph node metastasis positive/negative	63 (33.5%)/125 (66.5%)
Stage I/II/III	99 (52.7%)/74 (39.4%)/14 (7.4%)^a^

^1^Luminal (ER+ /HER2−).

^2^HER2 (HER2 +).

^3^TN (ER−, PR−, HER2−).

^a^Unknown for one case.

### Association of reduced E-cadherin expression with location, and relationship between reduced E-cadherin expression and LN metastasis

**Figure 2:**
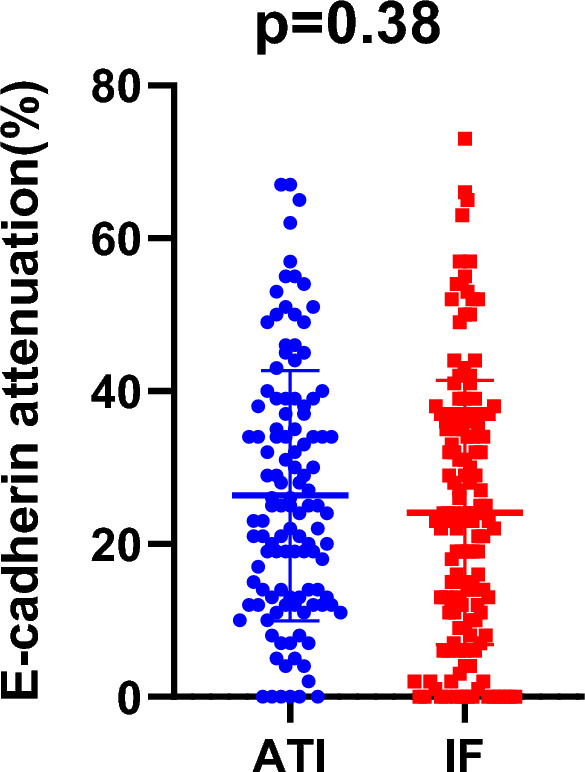
Relationship of reduced E-cadherin expression between adipose tissue invasion (ATI) and invasive front (IF) area (*p* = 0.38).

Of the 188 patients, E-cadherin expression was evaluated in 117 ATI cases and 140 IF cases. Forty cases were excluded from the IF measurement as the IF area was absent in cases where the tumor was surrounded by adipose tissue. The median of reduced E-cadherin expression in ATI and IF area was 29 and 24%, respectively, and no significant difference was seen between the ATI and IF groups (Fig. 2, ATI vs. IF,* p* = 0.38). Reduced E-cadherin expression in the ATI area was positively correlated with that of the IF area (correlation coefficient: 0.41, *p* < 0.01).

**Figure 3:**
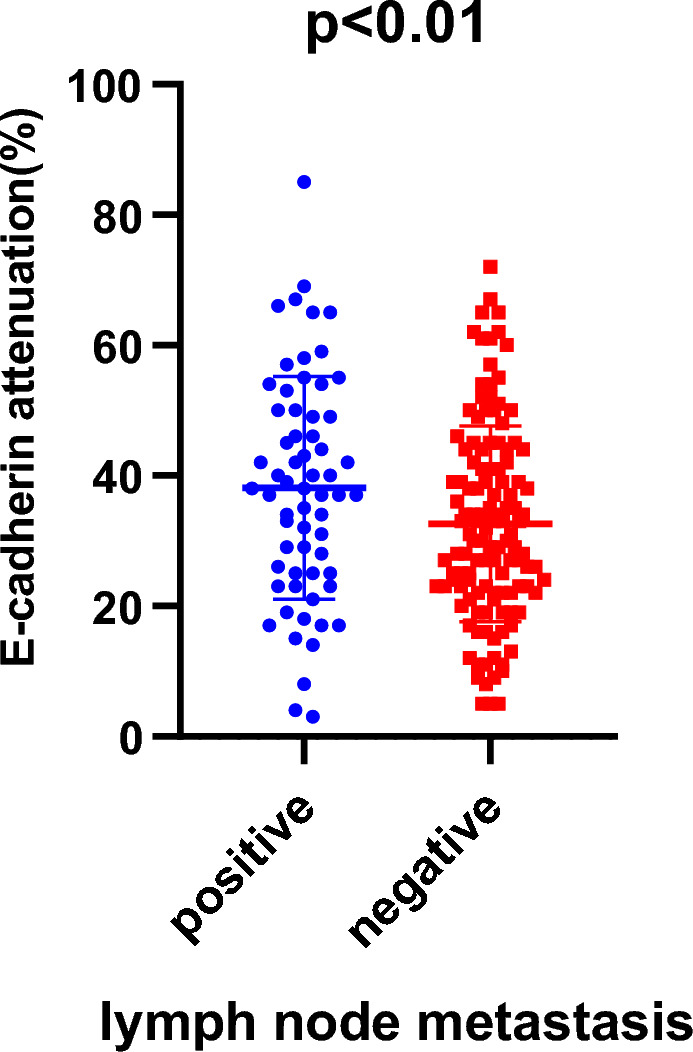
Relationship between reduced E-cadherin expression and lymph node (LN) metastasis. LN metastasis-positive patients had more E-cadherin reduction than negative patients (*p* < 0.01).

At the representative value, which is the maximum reduced E-cadherin expression at both ATI and IF areas, E-cadherin expression was significantly reduced in patients with LN metastasis than in those without (38% [3–85%] vs. 32% [5–72%], respectively; *p* < 0.01, Fig. 3).

### Association of LN metastasis and reduced E-cadherin expression

The LN metastasis-positive patients had high nuclear grade (*p* = 0.02), lymphovascular invasion (*p* < 0.01), and vessel invasion (*p* < 0.01), bigger tumor size (*p* < 0.01), and reduced E-cadherin expression (*p* < 0.01) than the LN metastasis-negative patients, in univariate analysis (Table 2). Lymphovascular invasion (*p* < 0.01) and tumor size (*p* < 0.01) were independent associatedly factors with respect to LN metastasis, and E-cadherin expression tended to be an independent prognostic factor for LN metastasis (*p* = 0.06) in multivariate analysis (Table 2).

**Table 2:** Relationship between LN metastasis and clinicopathological characteristics of patients with invasive breast cancer.

	Univariate		*p*-value	Multivariate	*p*-value
Characteristics	With LN metastasis (n = 63)	Without LN metastasis (n = 125)		Odds ratio (95% CI)	
Median age (range)	56 (31–90)	60 (32–92)	0.07	–	–
Tumor size (cm)^※^	2.2 (0.4–6.5)	0.6 (0.6–5)	< 0.01	2.06 (1.31–3.25)	< 0.01
ATI positive/negative	49/14	82/43	0.09	–	–
Nuclear grade 1, 2/3	25/28	83/42	0.02	1.23 (0.56–2.73)	0.60
Lymphovasucuar invasion positive/negative	54/9	40/85	< 0.01	0.12 (0.05–0.29)	< 0.01
Vessel invasion positive/negative	51/12	64/61	< 0.01	0.60 (0.25–1.45)	0.26
Reduced E-cadherin expression^a^	0.35 (0–0.73)	0.22 (0–0.54)	< 0.01	8.50 (0.92–78.19)	0.06

^a^Median (range).

### Prediction of survival by reduced E-cadherin expression

**Figure 4:**
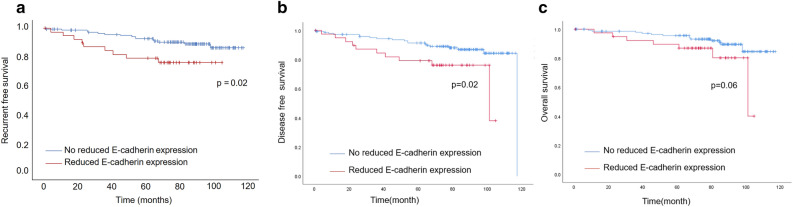
Survival analysis of reduced E-cadherin expression in patients with breast cancer. Relationship between reduced E-cadherin expression and (**a**) recurrence-free survival (RFS) (*p* = 0.02), (**b**) disease-free survival (DFS) (*p* = 0.02) and (**c**) overall survival (OS) (*p* = 0.06).

The cut-off value of reduced E-cadherin expression to predict RFS was 41% (area under the curve [AUC] = 0.563; 95% confidence interval [CI] = 0.441–0.685). Using 41% E-cadherin attenuation as the cut-off value, 40 and 148 patients were allocated to the group with reduced E-cadherin expression and without reduced E-cadherin expression, respectively (Table 3). The group with reduced E-cadherin expression had more frequent LN metastasis and RFS events, bigger tumor size, and increased HER2 subtype than the group without reduced E-cadherin expression (Table 3, Table S1). Moreover, patients with reduced E-cadherin expression demonstrated significantly shorter RFS and DFS intervals and tended to have shorter OS than patients without reduced E-cadherin expression (RFS: *p* = 0.02, Fig. 4a, DFS: *p* = 0.02, Fig. 4b, OS: *p* = 0.06, Fig. 4c).

**Table 3:** Comparison of clinicopathological factors with or without reduced E-cadherin expression.

Characteristics	Reduced E-cadherin expression group (n = 40)	Without reduced E-cadherin expression group (n = 148)	*p*-value
Median age (range)	55 (35–92)	60 (31–92)	0.22
Tumor size (cm)	1.95 (0.6–6.2)	1.5 (0.3–6.5)	< 0.01
ATI positive/negative	32/8	99/49	0.11
Nuclear grade 1/2/3	18/6/16	63/31/54	0.70
Lymphovascular invasion positive/negative	25/15	69/79	0.12
Vessel invasion positive/negative	28/12	87/61	0.20
ER positive/negative	37/3	133/15	0.62
PgR positive/negative^a^	34/6	118/29	0.50
HER2 positive/negative^a^	13/26	24/124	0.02
Subtype luminal/HER2/TN	24/13/2	114/24/10	0.06
LN metastasis positive/negative	22/18	41/107	< 0.01
Stage I/II/III	15/20/5	84/54/9	0.07
RFS events positive/negative	9/31	15/133	0.04

^a^Unknown for one case.

## Discussion

We quantitatively analyzed reduced E-cadherin expression using immunohistochemistry and examined the association of reduced E-cadherin expression with ATI, LN metastasis, and prognosis. Three major findings were obtained. First, reduced E-cadherin expression was seen at the tip of the tumor regardless of the site of ATI. Second, reduced E-cadherin expression was observed in patients with LN metastasis than in those without, and reduced E-cadherin expression was an independent predictive factor with respect to LN metastasis. Third, patients with reduced E-cadherin expression had worse RFS and DFS than those without.

Adipocytes are not only found adjacent to breast cancer cells, but also play an active role in the entire process of cancer development, progression, metastasis, and treatment response^14^. Aromatase is abundant in adipose tissue and promotes hormone-dependent breast cancer growth^15^. Aromatase inhibitors constitute the standard treatment for post-menopausal women with hormone receptor-positive breast cancer. Crosstalk between adipocytes and proximal cancer cells can lead to changes in the function and phenotype of both cell types; the interactions actively alter the tumor microenvironment^16^. Recently, however, some investigators highlighted the striking effects of adipocytes on MCF-7, T47D, and MDA-MB-231 human breast cell lines, including enhanced cell migration and invasion^17^. However, we did not find any significant difference in reduced E-cadherin expression between ATI-negative and -positive patients. The reason for this could be that co-culture with breast cancer cells was not exactly the same as tumor tissue invasion into adipose tissue. Even if the tumor did not invade the adipose tissue, adipokines produced by adipocytes may have an effect on the tumor. ATI may promote tumor progression without reducing E-cadherin expression, or alteration of epithelial phenotype, or regulation by other proteins and pathways.

A meta-analysis comprising retrospective studies of various methods of assessment, such as E-cadherin-positive cell percentages and the grading of E-cadherin-positive cells combined with staining intensity, reported that low E-cadherin expression was significantly associated with LN status (odds ratio 1.55, 95% CI 1.15–2.10)^4^. The definition of E-cadherin attenuation is important, with no significant association with lymph node metastasis in reports of ≥ 10% positive cells as E-cadherin positive^18^. Furthermore, reduced E-cadherin expression was not associated with DFS and OS in some studies in the aforementioned meta-analysis, but overall, the two were significantly associated (OS; HR 1.79, 95% CI 1.41–2.27, DFS; HR 1.62 95% CI 1.31–1.99)^4^. Previous reports focusing on subtypes have shown the association between E-cadherin downregulation and poor prognosis in TNBC^19^. Although this study mainly focused on luminal subtypes, we clarified a relationship between E-cadherin downregulation and poor prognosis in luminal-type breast cancer. Thus, reduced E-cadherin expression is a clear prognostic factor in breast cancer; however, it has not yet been applied to clinical practice. The reasons for this are that previous reports have been semi-quantitative and insufficiently objective and that assessment methods have varied. In our study, we used the free and downloadable ImageJ to quantitatively determine the reduced E-cadherin expression with objectivity. In addition, all previous reports have defined E-cadherin expression in each case, whereas the present study focused on heterogeneity within the tumor, and controls were placed in the same tumor to determine the reduced E-cadherin expression at the tumor margins. We speculated that tumor transformation and reduced E-cadherin expression could be important for tumor progression, since lobular carcinoma, characterized by the complete absence of E-cadherin, did not show worse survival than invasive ductal carcinoma. Therefore, quantitative measurements using ImageJ showed that patients with reduced E-cadherin expression of approximately 40% or more had a poor prognosis.

Our study had a few limitations. First, it had a small sample size and was a single-center retrospective study. Second, we used a microscope to visually select the areas of strongest E-cadherin expression as controls and where E-cadherin was attenuated at the tumor margins. This is somewhat less objective. Third, preoperative chemotherapy was excluded and only a few TNBC, HER2-positive, and high-stage patients were included. Therefore, we could not have enough cases to analyze according to each subtype, and the results of the study mainly apply to luminal-type breast cancer.

In conclusion, our study showed that reduced E-cadherin expression is not specific to the ATI area. Patients with reduced E-cadherin expression showed LN metastasis more frequently than those without metastasis. Reduced E-cadherin expression was associated with LN metastasis and with worse RFS in those with LN metastasis than in those without. Further quantitative analysis methods, such as using artificial intelligence, need to be established and validated so that reduced E-cadherin expression can be used as a prognostic factor in clinical practice.

## Supplementary Information

Supplementary Table S1.

Supplementary Figure S1.

## Data Availability

The datasets generated and analyzed during the current study can be provided by the corresponding author upon reasonable request.

## Abbreviations

ATI: Adipose tissue invasion
LN: Lymph node
RFS: Recurrence-free survival
TNBC: Triple-negative breast cancer
IF: Invasive front
DFS: Disease-free survival
OS: Overall survival
