# Supplementary material for: Association of quantitative analysis of intratumoral reduced E-cadherin expression with lymph node metastasis and prognosis in patients with breast cancer
Source: Sci Rep. 2023 Jun 27;13:10434. doi: 10.1038/s41598-023-37012-4 (PMC10300190; doi:10.1038/s41598-023-37012-4)
Supplement: Supplementary file 1 — Supplementary Table S1. [file 41598_2023_37012_MOESM1_ESM.docx]

Table 4 Odds ratios and 95% confidence intervals from bivariate and multivariable logistic regression model

predicting the likelihood that mothers in Jimma Arjo woreda having delayed initiation of breastfeeding, 2009

Supplementary Table S1: Odds ratio for association between reduced E-cadherin and clinicopathological characteristics

Odds ratios and 95% confidence intervals from bivariate and multivariable logistic regression model

predicting the likelihood that mothers in Jimma Arjo woreda having delayed initiation of breastfeeding, 2009

Odds ratios and 95% confidence intervals from bivariate and multivariable logistic regression model

predicting the likelihood that mothers in Jimma Arjo woreda having delayed initiation of breastfeeding, 2009

| Characteristics | Odds ratio (95% CI^a^) |
| --- | --- |
| Median age | 0.98 (0.96–1.01) |
| Tumor size | 1.37 (1.01–1.85) |
| ATI^b^ positive/negative | 2.21 (0.91–5.37) |
| Nuclear grade 3/1, 2 | 1.29 (0.63–2.67) |
| Lymphovascular invasion positive/negative | 1.71 (0.83–3.52) |
| Vessel invasion positive/negative | 1.49 (0.70–3.17) |
| ER^c^ positive/negative | 2.3 (0.51–10.42) |
| PgR^d^ positive/negative^※^ | 1.66 (0.60–4.63) |
| HER2 positive/negative^※^ | 2.02 (0.89–4.60) |
| Lymph node metastasis positive/negative | 2.41 (1.17–4.98) |
| Stage II, III/I^※^ | 1.98 (0.96–4.10) |

^a^Confidence interval

^b^Adipose tissue invasion

^c^Estrogen Receptor

^d^Progesterone Receptor

^※^Unknown for one case
