# Supplementary material for: Association of quantitative analysis of intratumoral reduced E-cadherin expression with lymph node metastasis and prognosis in patients with breast cancer
Source: Sci Rep. 2023 Jun 27;13:10434. doi: 10.1038/s41598-023-37012-4 (PMC10300190; doi:10.1038/s41598-023-37012-4)
Supplement: Supplementary file 2 — Supplementary Figure S1. [file 41598_2023_37012_MOESM2_ESM.pptx]

## Slide 1
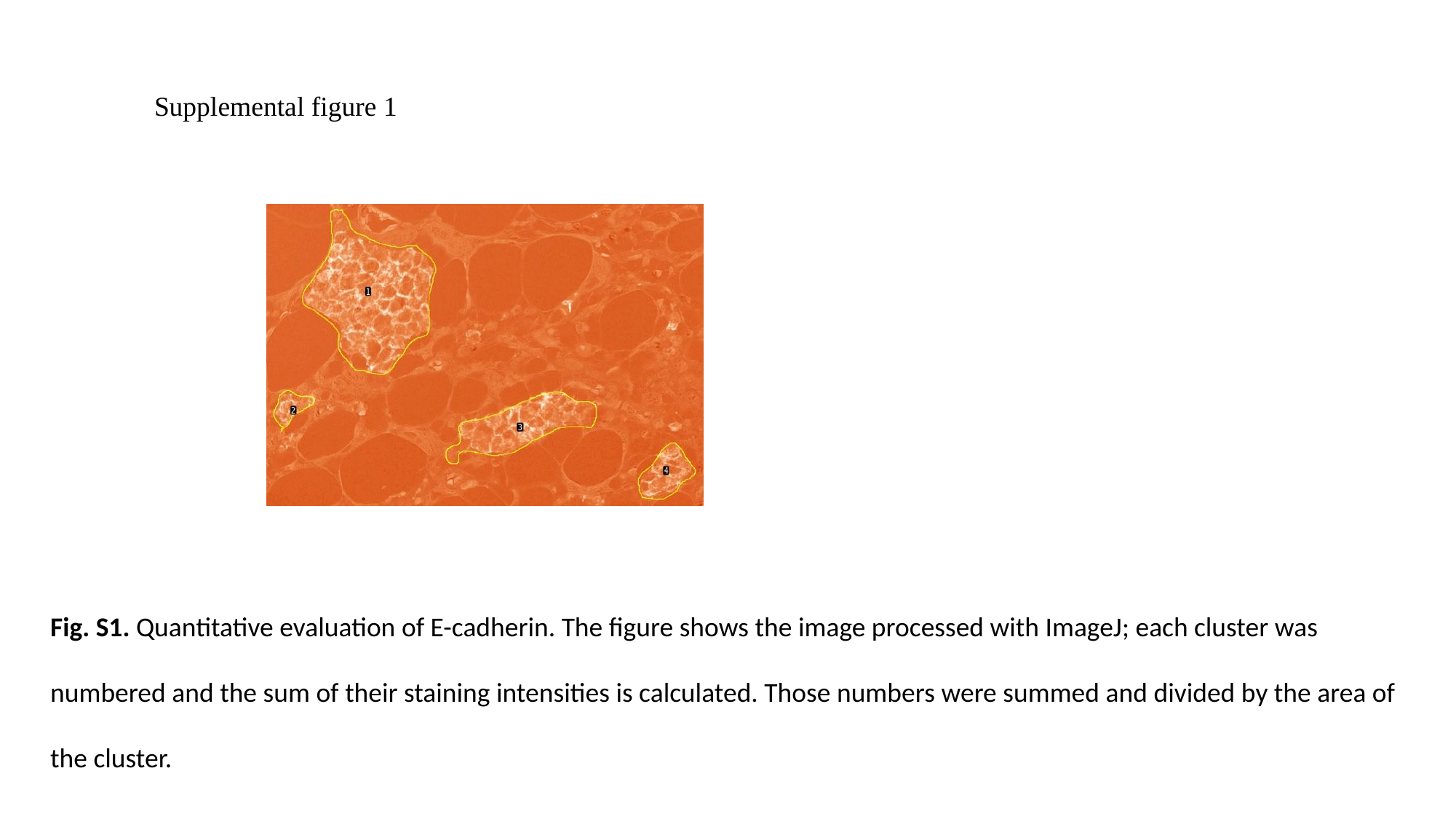

Supplemental figure 1
Fig. S1. Quantitative evaluation of E-cadherin. The figure shows the image processed with ImageJ; each cluster was numbered and the sum of their staining intensities is calculated. Those numbers were summed and divided by the area of the cluster.
